# Accelerometer Triad Calibration for Pole Tilt Compensation Using Variance Based Sensitivity Analysis

**DOI:** 10.3390/s20051481

**Published:** 2020-03-08

**Authors:** Tomas Thalmann, Manuel Zechner, Hans Neuner

**Affiliations:** Department of Geodesy and Geoinformation, TU Wien, 1040 Vienna, Austria; manuel.zechner@geo.tuwien.ac.at (M.Z.); hans.neuner@geo.tuwien.ac.at (H.N.)

**Keywords:** MEMS, IMU, accelerometer, leveling, tilt compensation, calibration, sensitivity analysis

## Abstract

In Engineering Geodesy, most coordinate frames are aligned with the local vertical. For many measurement tasks, it is therefore necessary to manually (or arithmetically) align sensors or equipment with the local vertical, which is a common source of errors and it is very time consuming. Alternatively, accelerometer triads as part of inertial measurement units (IMUs) are used in several applications for horizon leveling. In this contribution we analyze and develop a method to use accelerometer triads for pole tilt compensation with total stations. Several triad sensor models are investigated and applied in a calibration routine using an industrial robot arm. Furthermore a calibration routine to determine the orientation of the IMU mounted on the pole is proposed. Using variance based sensitivity analysis we investigate the influence of different model parameters on leveling and pole tilt compensation. Based on this inference the developed calibration routines are adjusted. The final evaluation experiment shows an RMS of 2.4 mm for the tilt compensated measured ground point with tilts up to 50 gon.

## 1. Introduction

An IMU (Inertial Measurement Unit) consists of tri-axial accelerometers, tri-axial gyroscopes and sometimes tri-axial magnetometers. A lot of research has been done about IMUs in fields of aerospace, navigation and robotics for several years. This is because of some unique and beneficial characteristics (compare, e.g., Reference [1] or Reference [2])—high temporal resolution, orientation estimation, high short term accuracy and unlimited availability independent from exterior environment.

In the early beginnings of IMU technology it was both rather expensive and unhandy in size [3]. Size, cost and power consumption have been dramatically reduced due to recent developments of MEMS (Microelectromechanical systems) [4]. This led to an even broader scope of applications and accelerated research [3].

Initially mainly used in navigation tasks, IMUs are now used in several applications for example, Augmented Reality, Indoor- and Smartphone navigation, Robotics and Mobile Mapping Systems. MEMS IMUs in particular are nowadays not only used for mobile mapping and navigation tasks by the geodetic community. Due to its unlimited measuring range compared to conventional inclination sensors, accelerometer horizon leveling attracted interest. Accelerometer horizon leveling means computation of two angles roll $\phi_{nb}$ and pitch $\theta_{nb}$ making use of accelerometer triads sensing the local gravity vector. In the following we use the term leveling instead of horizon leveling for the purpose of readability—not to be confused with leveling as a method for determination of height differences. Lately, accelerometer leveling has been used for deformation monitoring [5,6] and frequency analysis of vibrations, for example, References [7,8].

Another usecase of leveling with MEMS IMUs is tilt compensation of GNSS (Global Navigation Satellite System) poles, see Reference [9] or Reference [10]. Generally leveling is used in navigation and pose estimation during unaccelerated phases to stabilize attitude and to compensate gyroscope drifts. These direct measurements of the two tilt angles $\phi_{nb}$ and $\theta_{nb}$ are fed into IMU strapdown computation or a Kalman Filter for sensor fusion.

**Figure 1:**
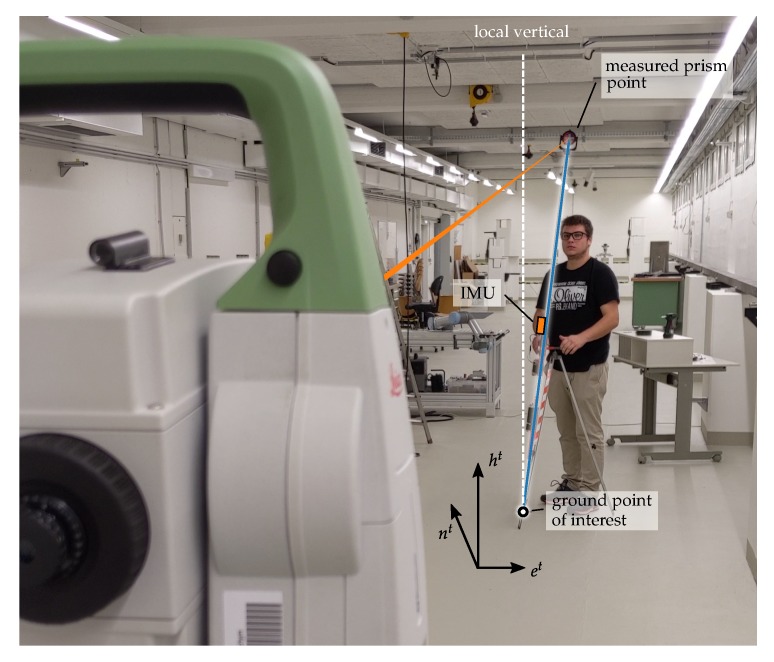
Illustration of pole tilt compensation for total stations. The ground point of interest w.r.t. the local geodetic frame is computed from the measured prism point using the estimated tilt from a pole mounted inertial measurement unit (IMU).

In Engineering Geodesy it is very often required to manually align instruments, sensors or measurement equipment with the local vertical defined by gravity. For 3D-point-measurements using a total station and a pole mounted prism, both need to be leveled using a circular level accurate to 6 arc minutes (see e.g., Reference [11]). Six arc minutes correspond to 2.6 mm horizontal position error at a height of 1.5 m. Modern total stations use liquid based tilt compensators to correct residual tilts. These sensors have inert measurement properties and a very limited measuring range [12]. For a prism pole no residual tilt correction sensor is available and errors up to 3 mm are easily introduced. Furthermore the leveling of a pole is the most time consuming part of measuring 3D-point-coordinates. The novel idea in this contribution is to bring the concept of IMU based leveling to the prism pole (see Figure 1), similar to what Reference [9] did for a GNSS pole. Advanced requirements on the accuracy of pole tilt compensation arise with the increased accuracy of a total station compared to GNSS. In this contribution we show how a calibration for such a system can be performed and we analyze which parameters are important. The accelerometer triad sensor models have to cope with the advanced accuracy demands. Extensive research has been carried out in the scientific community concerning IMU sensor models. The choice of the inertial instrument error model depends on the application/use-case and on the effect on the derived quantities [13] (p. 574f). Two basic categories of calibration approaches are distinguished: online and pre-calibration. In the first approach, parameters of the IMU error model (see Section 2.1) are estimated in real time using sensor fusion (e.g., Kalman Filter) with external observations, for example, GNSS-IMU integration. The deterministic observability of such state parameters depends on the user dynamics [13] (p. 574f) and is not applicable in static applications. In addition, pre-calibration should be preferred, due to the higher noise level of MEMS sensors, since

1. the possibilities to reduce noise in static environment through time-averaging, and 
2. the danger of vibrations overlaying systematics in kinematic applications.

Nevertheless, at least sensor biases should always be estimated online, since these parameters highly depend on temperature and might change significantly over time [14,15].

Two groups of pre-calibration methods referring to calibration setup can be found in literature. The first depends on additional equipment like a reference sensor (e.g., aviation grade IMU, rate tables [16,17,18,19], or optical 6DoF-tracking [20]) and is supposed to be executed in the laboratory. Such high precision equipment is expensive or might not be available [21] and is not economical for low-cost MEMS sensors [22]. We summarize these approaches as equipment-aided calibrations.

The other group of approaches targets suitable methods for in-field calibration. These methods mostly rely on gravity and should be feasible for end users. Reference [23] first introduced the accelerometer calibration using the property—the magnitude of the static acceleration measured must equal that of the gravity. This group is referred to as gravity-based approaches. Methods based on this property have in common, that gravity (g) is measured in multiple quasi-static positions (attitudes). Extensive research has been carried out, differing in number of positions and the underlying estimated error models. A summary can be found in Table 1.

In this contribution we investigate different accelerometer triad error models utilizing a 6-joints industrial robot. In order to compensate the pole tilt, additional mounting parameters of the triad w.r.t. the pole need to be estimated.

The remainder of this paper is organized as follows. In Section 2 we describe the various models of accelerometer triads, gravity-based leveling and pole tilt compensation. In the following Section 3, we show how the triad model parameters are calibrated and how the mounting parameters are estimated. In Section 4 we investigate calibration and mounting parameters concerning their contribution to the uncertainty of the final tilt compensation. Here variance based sensitivity analysis is used to identify and improve important parameters. The results of the calibration and a final evaluation experiment are presented in Section 5, whereas Section 6 concludes this contribution.

## 2. Methodology

**Figure 2:**
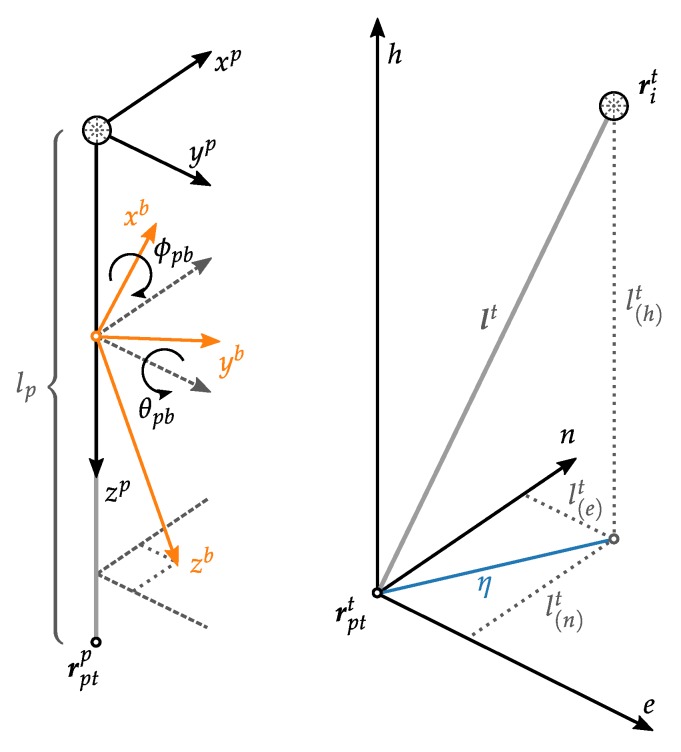
(**left**) The IMU body frame (${}^{b}$) and the pole frame (${}^{p}$) related by the mounting parameters $\phi_{pb}$ and $\theta_{pb}$. (**right**) Visualization of the tilted pole and the components of the pole vector in the *t-frame*. Including the horizontal displacement η due to tilting in blue, compare Figure 1.

To reduce the measured prism to the ground point for a tilted pole, it is necessary to know the orientation of the pole. This involves several coordinate frames (compare Figure 1 and Figure 2) accumulating in the transformation from pole frame ${}^{p}$ to the *local geodetic frame*${}^{t}$ (the frame of total station measurements). First we define the coordinate system of the pole (*pole frame* denoted by ${}^{p}$) so that the origin is placed at the prism, $z^{p}$ points downwards to the tip, $x^{p}$ lies in the plane defined by the IMU axis $x^{b}$ and $z^{b}$; $y^{p}$ completes the right-handed coordinate system. The sensitive axes of the 3 accelerometers form the *sensor frame* denoted by ${}^{s}$. The calibrated forces of the accelerometer triad refer to the *body frame* (${}^{b}$) of the IMU. The process of leveling determines the rotation of the *body frame* w.r.t. the local *navigation frame* (aligned with local vertical) denoted by ${}^{n}$.

Making use of these frames, pole tilt compensation involves three main computation steps:

1. Application of an appropriate tri-axis accelerometers sensor model to derive calibrated specific force measurements, which is in fact transformatoion from *s-frame* to *b-frame*. (Section 2.1)
2. In unaccelerated motion the sensed earth gravity can be used to compute roll $\phi_{nb}$ and pitch $\theta_{nb}$ attitudes w.r.t. the *n-frame*.
3. In combination with an externally estimated yaw $\psi_{nb}$ angle, the pole length $l_{p}$ and the mounting parameters, the components of the askew pole w.r.t. the *t-frame* is computed.

### 2.1. Sensor Models

Several sensor models can be found in literature. They differ mainly in the modeled error parameters. The basic model for the measured accelerometer outputs (measured forces) denoted by $f_{s}={\left[\begin{array}{ccc} f_{x,s} & f_{y,s} & f_{z,s} \end{array}\right]}^{\;T}$ proposed by Reference [23] is:

(1) $$\begin{array}{c} f_{s}=S_{f}\;f^{a}+b_{f}+v_{f}\;, \end{array}$$

where $f^{a}={\left[\begin{array}{ccc} f_{x}^{a} & f_{y}^{a} & f_{z}^{a} \end{array}\right]}^{\;T}$ is the calibrated force vector, $b_{f}={\left[\begin{array}{ccc} b_{x} & b_{y} & b_{z} \end{array}\right]}^{\;T}$ is the offset or biases vector and

(2) $$\begin{array}{c} S_{f}=\left[\begin{array}{ccc} 1+s_{x} & 0 & 0\\ 0 & 1+s_{y} & 0\\ 0 & 0 & 1+s_{z} \end{array}\right] \end{array}$$

is the scale factor diagonal matrix and $v_{f}$ is the accelerometer random noise.

**Figure 3:**
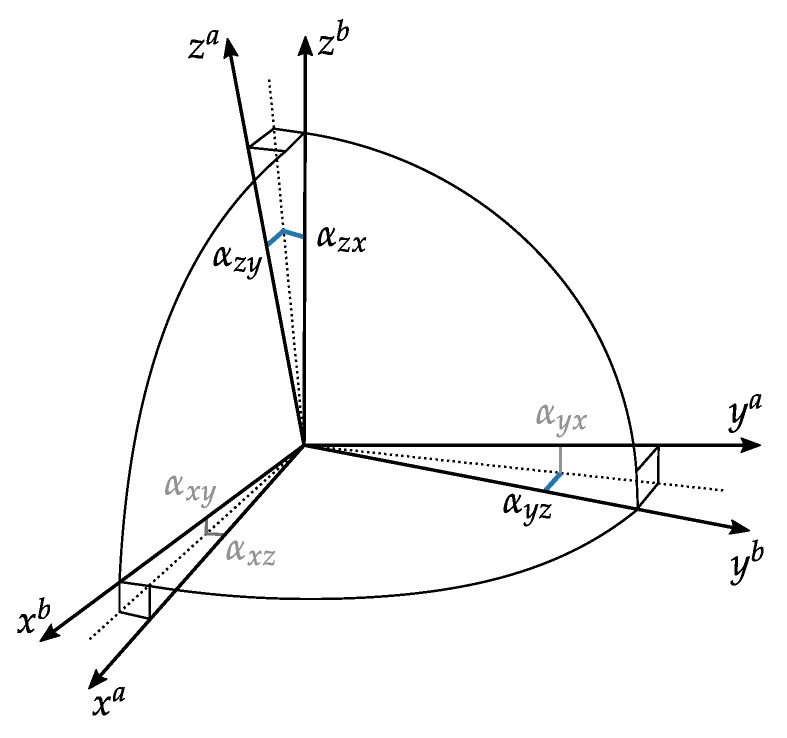
The non-orthogonal sensitivity axes ${}^{a}$ can be transformed to the orthogonal body frame ${}^{b}$ by 6 small angles (after Reference [16]).

The calibrated forces $f^{a}$ refer to the three accelerometer sensitivity axes, thus denoted by ${}^{a}$. Ideally these axes should be orthogonal, but due to imprecise manufacturing this is most likely not the case. Therefore, Reference [16] extended their model to account for this non-orthogonality of the sensor sensitivity axis by introducing

(3) $$\begin{array}{c} f^{b}=T_{a}^{b}\;f^{a}\;,\;\mathrm{with}\;T_{a}^{b}=\left[\begin{array}{ccc} 1 & -\alpha_{yz} & \alpha_{zy}\\ \alpha_{xz} & 1 & -\alpha_{zx}\\ -\alpha_{xy} & \alpha_{yx} & 1 \end{array}\right]\;, \end{array}$$

which transforms the sensitivity axes to the orthogonal body or IMU-frame (denoted by ${}^{b}$) by use of 6 parameters. Here these parameters can be interpreted as *small* angles, where $\alpha_{ij}$ is the rotation of the *i*-th axis around the *j*-th body axis, compare Figure 3. If both rotations about one axis are equal, for example, $\alpha_{xz}=\alpha_{yz}$ for all rotation axes, Equation (3) becomes a skew-symmetric matrix, which corresponds to the well known small angle approximation rotation matrix.

Defining the body frame so that *x*-axes coincide and $y^{b}$-axis lies in the plane spanned by $x^{a}$ and $y^{a}$ Equation (3) reduces to (compare Figure 3):

(4) $$\begin{array}{cc} f^{b}=T_{a}^{b}\;f^{a}\;,\; & \mathrm{with}\;T_{a}^{b}=\left[\begin{array}{ccc} 1 & -\alpha_{yz} & \alpha_{zy}\\ 0 & 1 & -\alpha_{zx}\\ 0 & 0 & 1 \end{array}\right]\\ \; & \mathrm{and}\;{T_{a}^{b}}^{-1}=T_{b}^{a}=\left[\begin{array}{ccc} 1 & \alpha_{yz} & \alpha_{zx}\;\alpha_{yz}-\alpha_{zy}\\ 0 & 1 & \alpha_{zx}\\ 0 & 0 & 1 \end{array}\right]\;. \end{array}$$

This gives a 9-parameter model by extending Equation (1) with Equation (4) comprising three additional non-orthogonality parameters $\alpha_{f}={\left[\begin{array}{ccc} \alpha_{zx} & \alpha_{zy} & \alpha_{yz} \end{array}\right]}^{T}$:

(5) $$\begin{array}{c} f_{s}=S_{f}\;T_{b}^{a}\;f^{b}+b_{f}+v_{f}\;. \end{array}$$

### 2.2. Leveling

Following equations are used for accelerometer leveling [13], which describe the orientation of the IMU body frame ${}^{b}$ with respect to the local navigation frame denoted by ${}^{n}$. Euler angles are used to describe the attitude using roll $\phi_{nb}$, pitch $\theta_{nb}$ and yaw $\psi_{nb}$ rotations.

(6) $$\begin{array}{cc} \phi_{nb} & ={\mathrm{atan}}_{2}\left(-f_{y}^{b},-f_{z}^{b}\right)\\ \theta_{nb} & =\mathrm{atan}\left(\frac{f_{x}^{b}}{\sqrt{{f_{y}^{b}}^{2}+{f_{z}^{b}}^{2}}}\right) \end{array}.$$

Note that arctan2() must be used for roll computation, but if limiting tilting to the upper half sphere it can be replaced by arctan().

The accuracy of this leveling process depends on the model parameters for accelerations and accelerations noise. For example, a 1 mrad roll and pitch accuracy is obtained from accelerometers accurate to $10^{-3}$ g. However, disturbing motions such as vibrations or human activity influences the leveling process. In case this motion averages out over time, its effects may be reduced simply by time-averaging [13]. This is applied in all experiments in the remainder of this contribution, since human shaking during pole handling is relevant for the application regarded in this case. Furthermore one has to consider present auto-correlation and cross-correlation in the specific force measurement data.

### 2.3. Pole Tilt Compensation

For coherent pole tilt compensation it is necessary to know the exact relation between the IMU *b-frame* and the poles *p-frame*. The IMU is placed somewhere on the pole rotated by the Euler angles $\psi_{pb}$ which define the rotation from *b-frame* to *p-frame* (see Figure 2). These angles can be converted to a rotation matrix or coordinate transformation matrix $C_{b}^{p}$:

(7) $$\begin{array}{c} \psi_{pb}=\left[\begin{array}{c} \phi_{pb}\\ \theta_{pb} \end{array}\right]\to C_{b}^{p}=C_{b}^{p}\left(\phi_{pb},\theta_{pb}\right) \end{array}\;,$$

where the definition of rotation axes and rotation sequence is shown in Appendix A. The pole length $l_{p}$ is the distance from prism center to the pole tip and can be expressed in pole coordinates as:

(8) $$l^{p}=\left[\begin{array}{c} 0\\ 0\\ l_{p} \end{array}\right].$$

With the rotation of the pole frame to the local horizontal navigation frame (${}^{n}$) defined by $\psi_{np}$ or $C_{p}^{n}$, the tilted pole can be converted to horizontal *n-frame* by (see also Figure 2):

(9) $$\begin{array}{cc} l^{n} & =C_{p}^{n}l^{p}=C_{b}^{n}C_{p}^{b}l^{p}=C_{b}^{n}{C_{b}^{p}}^{T}l^{p}=\\ \; & =C_{3}{\left(\psi_{nb}\right)}^{T}\;C_{b}^{n}\left(\phi_{nb},\theta_{nb}\right)\;C_{b}^{p}{\left(\phi_{pb},\theta_{pb}\right)}^{T}\;l^{p}\;, \end{array}$$

where $C_{3}\left(\psi_{nb}\right)$ must be determined from heading estimation, $C_{b}^{n}\left(\phi_{nb},\theta_{nb}\right)$ is computed by accelerometer leveling and $C_{b}^{p}\left(\phi_{pb},\theta_{pb}\right)$ describes the mounting and needs to be calibrated. For the notation using parentheses, please refer to Appendix A. The resulting $l^{n}$ then describes the coordinate components of the tilted pole w.r.t. the *n-frame*.

Finally the coordinates of the pole tip $r_{pt}^{t}$ (= the ground point) are computed from the measured prism point $r_{i}^{t}$:

(10) $$r_{pt}^{t}=r_{i}^{t}+R_{n}^{t}\;l^{n}\;,\;\mathrm{with}\;R_{n}^{t}=\left[\begin{array}{ccc} 0 & 1 & 0\\ 1 & 0 & 0\\ 0 & 0 & -1 \end{array}\right]\;,$$

where $R_{n}^{t}$ takes care of converting the *n-frame* to the local geodetic frame (${}^{t}$). The frames *p*, *b*, *n* are designed as NED-system, while the *t-frame* (= the total station system) is usually an ENU-system.

## 3. Calibration Procedure

The proposed calibration procedure here consists of two parts.

1. The first part involves a 6-joints industrial robot arm, holding the IMU under consideration to perform the tri-axial **accelerometer calibration**, referring to the sensor models in Section 2.1.
2. In the second part a total station is used to estimate the mounting parameters $\psi_{pb}$ introduced in Equation (7), Section 2.3. We call this sub-procedure **mounting estimation**.

For the first, the method is described in Section 3.1 to estimate the parameters of various sensor models introduced in Section 2.1. We investigate different subsets of parameters in Equation (5) giving the following models (denoted by $I_{i}$) listed below:

1. Bias only model ($f_{s}=f^{a}+b_{f}+v_{f}$) denoted by $I_{0}$
2. Bias and Scale 6 parameter model Equation (1) denoted by $I_{1}$
3. Full 9 parameter model Equation (5) denoted by $I_{2}$

These are estimated using the 24 position scheme proposed by Reference [26], see Section 3.1. For the mounting estimation, the IMU is mounted on a prism pole and the prism is tracked by a total station. By tilting the pole in different directions over a known ground point the mounting parameters are determined. The method is described in Section 3.2.

### 3.1. Accelerometers Calibration

The *gravity-based* approach uses the fact, that the norm of measured gravity must be independent from attitude of the IMU under quasi-static conditions [23]. As long as the position displacement does not exceed a certain limit the length of $g^{n}$ is constant. Apart from local gravity variation a change in longitude has no effect, a change in latitude means about 1 mm s^−2^ per 55 km. Theoretically the norm of the measured specific force must equal the norm of the gravity:

(11) $$∥f^{b}∥=∥g^{n}∥\;.$$

The disadvantage of these approaches is, that $g^{n}$ must be known. Generally, no exact measurement of $g^{n}$ is available, but values from theoretical models can be computed. Anyhow for applications exclusively concerned about leveling the length of $g^{n}$ does not matter, hence a length of 1 could have been used [29].

(12) $$\begin{array}{cc} \varphi (l+v,x) & =\sqrt{{f_{x}^{b}}^{2}+{f_{y}^{b}}^{2}+{f_{z}^{b}}^{2}}-g^{n}=0\\ \; & =∥f^{b}∥-∥g^{n}∥=∥T_{a}^{b}\;S_{f}^{-1}\;\left(f_{s}-b_{f}\right)∥-∥g^{n}∥=0\;. \end{array}$$

Model (12) can be used as the functional part of a Gauss-Helmert-Adjustment (GH) to estimate the different model parameters of the tri-axial accelerometers of Equation (5) by replacing $f^{b}$ with the corresponding model by inverting Equation (5). The scale matrix $S_{f}$ is easily invertible, since it is a diagonal matrix. For the application of Equation (5) it is also necessary, that $T_{a}^{b}$ is invertible, which is given by the fact, that triangular matrices are invertible if all diagonal elements are non-zero, which is the case for $T_{a}^{b}$. This is then solved using the well-known iterative nonlinear least squares algorithm, cf. Reference [30]. Since 9 parameters are to be estimated in Equation (5), at least 9 positions (=9 condition equations like (5)) are required [17]. However, a more sophisticated calibration scheme is necessary for reliable estimates of the parameters. Reference [26] proposed a 24-position scheme consisting of eight 50 gon rotations per accelerometer axis. This scheme distributes the measured $g^{n}$-vector evenly over the unit-sphere. The stochastic model is then made up of a block diagonal matrix $\Sigma_{ll}$, consisting of *n* (=number of positions) 3 × 3 matrices $\Sigma_{f}$ empirically determined. This is done by estimating the variance covariance matrix (VCM) using approx. 250 measurements during each stable position. Afterwards the variance of the mean is computed, since the epochs have been proven uncorrelated during auto-correlation investigations.

From a practical point of view, this calibration approach is also applicable in the field, without additional equipment and data collection can be done by hand. Others have used rotation tables or polyhedrons for IMU placement. In this contribution we use a 6-joints industrial robot arm as IMU carrier. This has some advantages: unlimited spectrum of possible attitudes, accurate reproducibility and time efficiency, since no human interaction is necessary.

### 3.2. Mounting Calibration

To estimate the mounting of the IMU on the pole, that is the rotation $\psi_{bp}$ between the pole system (*p-frame*) and IMU system (*b-frame*) we tilt the pole with the tip fixed on a coordinatively known ground point $r_{pt}^{t}$. Starting from Equation (10) the following condition must hold:

(13) $$r_{i}^{t}+R_{n}^{t}\;l^{n}-r_{pt}^{t}=0.$$

Denoting $r_{pt}^{t}-r_{i}^{t}$ by $l_{\prime }^{t}$ describing pole components measured by the total station, and $l^{n}$ as pole components measured by IMU, the condition can be rewritten as:

(14) $${R_{n}^{t}}^{T}\;l_{\prime }^{t}-l^{n}={\left[\begin{array}{c} n_{\prime }\\ e_{\prime }\\ d_{\prime } \end{array}\right]}^{n}-{\left[\begin{array}{c} n\\ e\\ d \end{array}\right]}^{n}=0.$$

To get rid of the additional yaw parameter $C_{3}\left(\psi_{nb}\right)$ in Equation (9), we consider only horizontal displacement $\eta =\sqrt{n^{2}+e^{2}}$ of the prism. Then condition, Equation (14) reduces to:

(15) $$\sqrt{n_{\prime }^{2}+e_{\prime }^{2}}-\sqrt{n^{2}+e^{2}}=\eta_{\prime }-\eta =0.$$

This way the attitude of the IMU $\psi_{nb}$ reduces to the leveling parameters $\phi_{nb},\;\theta_{nb}$, while the mounting parameters $\psi_{bp}$ already only consist of $\phi_{bp},\;\theta_{bp}$ because of the frame definitions described in Section 2. With Equation (15), the following condition equation can be defined for every prism position:

(16) $$\varphi \left(l+v,x\right)=\eta_{\prime }-\eta =\varphi \left(\underset{l}{\underset{︸}{e_{i}^{t},n_{i}^{t},e_{pt}^{t},n_{pt}^{t},f_{x}^{b},f_{y}^{b},f_{z}^{b}}},\overset{x}{\overset{︷}{\phi_{pb},\theta_{pb}}}\right)=0.$$

The parameters in this model are the roll $\phi_{pb}$ and pitch $\theta_{pb}$ from *p-frame* to *b-frame*. The east $e_{i}^{t}$ and north $n_{i}^{t}$ coordinates of the prism, measured by the total station form the observation vector together with the IMU accelerometer readings $f^{b}$. We have also designed the ground point ($e_{pt}^{t},n_{pt}^{t}$) as observations to account for the small gliding or moving of the pole tip while tilting the pole. The pole length $l_{p}$ is considered a non-stochastic quantity in this model. The pole is held static for a period of about 5 to 10 s using a three-legged clamp (see Section 5). A scheme of 40 positions with tilts of up to 50 gon is performed.

One can think of combining mounting estimation and accelerometer calibration into one single adjustment by replacing $f^{b}$ in Equation (16) with the according sensor model, for example, Equation (5). In theory, that would be the preferable approach. We have decided to split these two adjustments, because from a practical point of view, it is only possible to tilt the pole over a known ground point up to about 50 gon, whereas for the accelerometer calibration it is necessary to have rotations all over the sphere.

## 4. Variance Based Sensitivity Analysis

Variance based sensitivity analysis (VBSA) can be used to analyze the relations between input and output parameters of a model. The goals of sensitivity analysis are listed in References [31,32]:

1. model validation,
2. model optimization, and 
3. identification of important parameters.

**Figure 4:**
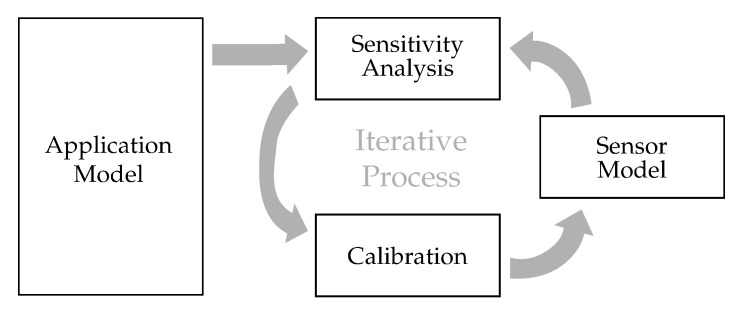
The idea of application-centric calibration optimization.

This concept was brought to the engineering geodesy context by References [32,33] and has been used in many studies since then (e.g., References [34,35]). Please refer to these references for details of the methodology and implementation. In this contribution VBSA is a powerful tool to analyze and optimize the calibration process under consideration of the target application. The basic idea is shown in Figure 4.

The sensor model is already given in Section 2.1 (5):

(17) $$f^{b}=S\left(b_{f},s_{f},\alpha_{f},f_{s}\right)\;,$$

which connects sensed specific forces $f_{s}$ to calibrated specific forces $f^{b}$ through the calibration parameters $b_{f},s_{f},\alpha_{f}$ (based on the model $I_{2}$, Section 3). It is not clear from the first sight, which calibration parameters are important for specific applications, in our case **leveling** or **pole tilt compensation**. For this purpose we investigate the influence of the calibration parameters on the outputs of the following application models:

(18) $$\phi_{nb},\theta_{nb}=A_{1}\left(f^{b}\right)=A_{1}\left(S\left(b_{f},s_{f},\alpha_{f},f_{s}\right)\right)\;\mathrm{for}\;\mathrm{leveling},\;\mathrm{and}$$

(19) $$l^{n}=A_{2}\left(S\left(b_{f},s_{f},\alpha_{f},f_{s}\right),\psi_{nb},\phi_{pb},\theta_{pb}\right)\;\mathrm{for}\;\mathrm{pole}\;\mathrm{tilt}\;\mathrm{compensation}.$$

The goal is to improve the calibration configuration in an efficient way to improve the output parameters of the application model in a sense of reduced variance. Our approach first analyzes the contribution of each input quantity on the output variance of the application model under consideration, for example, roll and pitch of model $A_{1}$. This can be done utilizing VBSA, computing the *total effect* $S_{T,i}^{j}$ for the *i-th* input measure, for example, $X=\left[\begin{array}{cccc} b_{f}^{T} & s_{f}^{T} & \alpha_{f}^{T} & f_{s}^{T} \end{array}\right]$ on the *j-th* element of $Y=\left[\begin{array}{cc} \phi_{nb} & \theta_{nb} \end{array}\right]$ by:

(20) $$S_{T_{i}}^{j}=\frac{E\left(\sigma_{\left(Y_{j}|X_{\neg i}\right)}^{2}\right)}{\sigma_{Y_{j}}^{2}}\;.$$

Here $\sigma_{Y_{j}}^{2}$ describes the variance of the output parameter and $\sigma_{\left(Y_{j}|X_{\neg i}\right)}^{2}$ describes the output variance that one would end up with if all other quantities except $X_{i}$ could be known or fixed. The total effect terms are computed using a special sample scheme for the correct generation (=simulation) of the conditional variances using the original nonlinear models. The simulation and analysis of correlated input is not considered so far. Sensitivity analysis for leveling is shown in Section 4.1. The calibration parameter with the highest effect is the one to be improved. A method to improve the calibration configuration for a GH-estimation of the calibration parameters taking these aspects into account is shown in Section 4.2. This is an iterative process, that can be repeated until the desired output variance of the application model is reached. This section is concluded by the sensitivity analysis of the pole tilt compensation (Section 4.3).

### 4.1. Leveling

**Figure 5:**
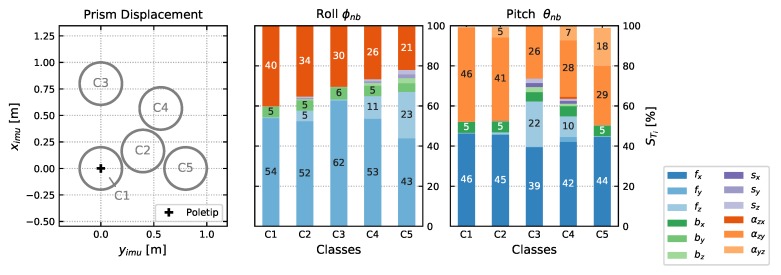
Total effect terms $S_{T_{i}}*100$ of IMU sensor model parameters on leveling for different pole tilts. The classes are defined by regions of prism displacement up to 50 gon tilt shown in the left subplot.

The total effect terms of the IMU sensor model parameters on the leveling output quantities Equation (18) $A_{1}$ are shown in Figure 5. The computation is based on the results of the IMU Calibration $I_{2}$ (see Section 5.1) and a $\sigma_{f_{s}}$ of 30 mm s^−2^ from empirical investigations of the IMU in use. This corresponds to a root noise density $S_{f_{s}}$ of 4.2 mg/$\sqrt{H}z$. Time averaging of forces over n=50 uncorrelated epochs is also considered. The theoretical standard deviation of tilt angles $\psi_{nb},\theta_{nb}$ from variance-covariance propagation is about 160 mgon with variations of ±3 mgon. The analysis is done for 5 classes, depending on the tilting and the resulting prism displacement. $C_{1}$ represents the approximately leveled case, $C_{2}$ results from a tilt of about 20 gon (=40 cm prism displacement) from vertical and $C_{3}$ to $C_{5}$ are tilted about 40 gon (=80 cm) in different directions. We have included only displacements for one quadrant, since we know from previous studies, that the influences are perfectly symmetric [36].

The following conclusions can be derived:

- For this IMU specifications, the raw observations $f_{s}$ make up about half of the output tilt variances with a small dependence on prism displacement.
- For smaller tilt angles < 30 [gon] *z*-accelerometer reading is inessential for both tilt angles, whilst *y*-accelerometer is dominant for roll $\phi_{nb}$ and *x*-accelerometer is dominant for pitch $\theta_{nb}$ computation. This makes sense, considering the corresponding rotation axes of the rotation angles (*x* for $\phi_{nb}$ and *y* for $\theta_{nb}$).
- Contribution of *z*-accelerometer readings increase with prism displacement (or tilt respectively) (compare $C_{1}$, $C_{2}$ with $C_{3}$, $C_{4}$, $C_{5}$)
- Concerning the calibration parameters, both bias and scale parameters have very low influence on the resulting parameter variance, whereas $\alpha_{zx}$ has a big share on the variance of $\phi_{nb}$ and $\alpha_{zy}$ on $\theta_{nb}$. This is plausible, since for example, $\alpha_{zx}$ nearly directly distort roll $\phi_{nb}$, since both are rotations about the *x*-axis, compare Figure 3.

Whilst we have no influence on the IMU specification from a methodogical point of view, except of buying a better one, we can focus on the two non-orthogonality parameters $\alpha_{zx},\alpha_{zy}$ to improve leveling performance, which cause up to almost 50% of the leveling uncertainty.

### 4.2. Improvement Strategy

With this conclusions from VBSA one can ask for an optimal or improved accelerometer calibration configuration. But not with the goal to improve the overall result (by means of minimal variance of all the parameters), instead search for a configuration to improve the most relevant parameters for the specific application, namely the non-orthogonality parameters $\alpha_{zx},\alpha_{zy}$ identified before in Section 4.1. Practically this means searching for calibration positions (in fact = pose), that maximize the sensitivity of measurements on the identified parameters.

For this purpose we developed an *Adjustment Sensitivity Algorithm* inspired by VBSA. Starting with the 24 position scheme proposed by Reference [26] (from now on denoted $L_{0}$) the expected VCM of parameters can be computed [37] (for matrix notation and computation please refer to Appendix B or Reference [37]):

(21) $$\Sigma_{\hat{x}\hat{x}}={\left(A^{T}{\left(B\;\Sigma_{ll}\;B^{T}\right)}^{-1}A\right)}^{-1}.$$

**Figure 6:**
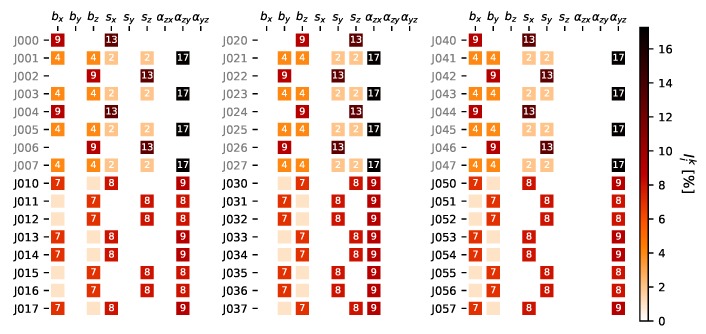
Improvement terms $I_{i}^{k}*100$ of the positions in $L_{c}$ on the resulting parameter variances of the initial 24 position scheme (positions already contained in $L_{0}$ are written in gray).

This is defined as the initial or starting VCM $\Sigma_{0}$. For every pose (i) from the improvement candidate set $L_{c}$ the resulting VCM $\Sigma_{i}$ is computed again according to Equation (21). This is the variance to be expected when adding position *i* to the 24 position scheme resulting in a 25 position scheme. In our case the candidate set was chosen to consist of the initial set $L_{0}$ and and extension set $L_{1}$ consisting of eight additional 25 gon rotations per axis, giving 24 additional positions. For the positions of $L_{0}$ the VCM $\Sigma_{i}$ indicates the resulting VCM, when measuring the respective position twice. For every position of $L_{c}$ the improvement by means of variance reduction can be computed by:

(22) $$I_{i}^{k}=1-\frac{\Sigma_{i}\left(k,k\right)}{\Sigma_{0}\left(k,k\right)}=1-\frac{\sigma_{k,i}^{2}}{\sigma_{k,0}^{2}}\;,$$

which is the relative improvement of the *i*-th candidate on the *k*-th parameter variance. The results for all improvement measures are shown in Figure 6. Now we can pick the positions with the biggest improvement on the target parameter and might repeat the procedure until a certain exit criterion is met. For a detailed description of this algorithm, please refer to Algorithm A1 in Appendix B.

The naming of the positions is defined as: J000 to J007 are rotations about horizontal *y*-axis from 0 gon (*x*-axis vertical) to 350 gon in 50 gon steps. J010 to J017 are the corresponding 25 gon to 375 gon rotations about horizontal *y*-axis. The positions J020 to J027 and J030 to J037 follow the same scheme about the *x*-axis and J040 to J047 and J050 to J057 about the *z*-axis. We can deduce from Figure 6, that $\alpha_{ij}$ is best estimated if the rotation axis *j* is horizontal and the rotated axis *i* (see Figure 3) forms a ±50 or ±150 gon angle with the gravity vector. These findings match the conclusions from Reference [38], who did this investigations prior to their estimation process.

For this calibration we picked the eight positions contributing the most on $\alpha_{zx}$ and $\alpha_{zy}$ and stopped, giving a resulting calibration scheme $I_{3}$ of 32 positions. For comparison the final evaluation investigations have also been done with a full calibration scheme $I_{4}$ consisting of $L_{0}$ and $L_{1}$ giving 48 positions.

### 4.3. Pole Tilt Compensation

**Figure 7:**
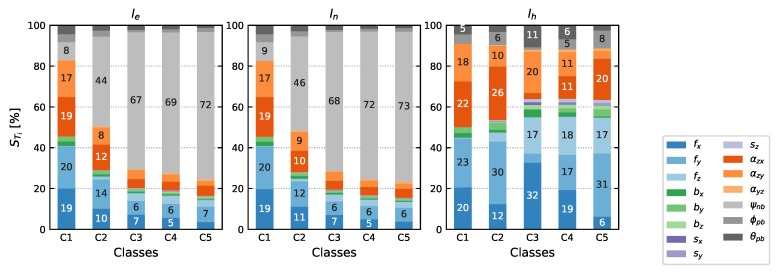
Total effect terms $S_{T_{i}}*100$ of IMU sensor model parameters, yaw $\psi_{nb}$ and the mounting parameters $\psi_{pb},\theta_{pb}$ on pole components for different pole tilts. The classes are defined similar to Figure 5.

The total effect terms $S_{T_{i}}$ on the pole components are shown in Figure 7. IMU specifications and sensor model uncertainties are chosen similar to Section 4.1, including uncertainties of the mounting parameters from calibration $M_{2}$ (Section 5.2) and a standard deviation $\sigma_{\psi }$ of the yaw $\psi_{nb}$ of 0.4 gon. The theoretical standard deviations, again from variance-covariance propagation, are 3.5 mm (horizontal components) and 0.08 mm (height) for $C_{1}$ and for example,  2.5 mm (east and height) and 6.6 mm (north) for $C_{5}$. It can be deduced, that again the two important non-orthogonality parameters $\alpha_{zx},\alpha_{zy}$ together make up almost 40% for near vertical pole tilts ($C_{1}$). With increasing horizontal displacement η the influence of the yaw estimation $\psi_{nb}$ increases and has a share of up to almost 75% of the total variance. The effect is similar for both *e* and *n* components, since we simulated yaw values $\psi_{nb}$ from a uniform distribution over the whole spectrum (−200 gon to +200 gon).

It’s important to keep in mind, that VBSA analyzes the stochastic influence of parameters, which does not necessarily agree with deterministic or functional influence. We will revisit this distinction in Section 5.1.

## 5. Experiments and Results

**Figure 8:**
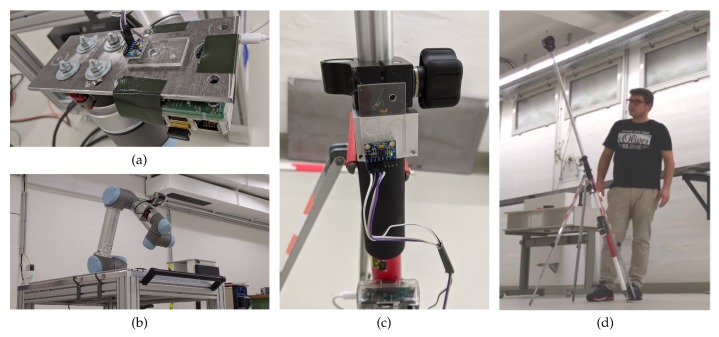
(**a**) the IMU *MPU6050* mounted on the robots end-effector. (**b**) the robot UR5 performing the calibration routine. (**c**) the IMU mounted at the prism pole. (**d**) One of the authors performing the mounting estimation.

To evaluate the proposed methodology a series of experiments were conducted. A consumer-grade MEMS (Micro-Electro-Mechanical Systems) IMU of type *MPU6050* of *InvenSens TDK* (see Figure 8) was used in all the experiments. For the IMU calibration an industrial robot arm *UR5* of *Universal Robots* was used as a carrier, see Figure 8b. For the mounting estimation and evaluation experiment a total station *TS 16* from *Leica Geosystems* was used.

First the IMU calibration described in Section 3.1 was done for the different sensor models $I_{0}$ to $I_{2}$ (Section 3) and customized positions schemes $I_{3}$ (32 positions) and $I_{4}$ (48 positions) based on $I_{2}$ (utilizing the same sensor model). During calibration every position was kept static for 5 s.

Secondly the mounting estimation introduced in Section 3.2 using the different sensor models $I_{i}$ giving the corresponding mounting models $M_{i}$. For this a 2.20 m pole was used with 40 evenly distributed positions up to 50 gon tilts. The pole is held static for a period of about 5 to 10 s using a three-legged clamp (see Figure 8d). The longest possible pole length has been chosen for this calibration, since an error in a mounting parameter has a bigger effect with longer poles.

Finally a pole tilt compensation evaluation experiment was performed, see Section 5.3.

### 5.1. IMU Calibration

The results of the different IMU calibrations are shown in Table 2, Table 3 and Table 4. For the first two models $I_{0}$ and $I_{1}$ the global test of the adjustment (see for example, Reference [39]) indicates discrepancy in the functional or stochastic model by test values of 765.8>1.6 and 8.4>1.6 respectively, see Table 5. The null hypotheses states that the posterior variance-factor $s_{0}^{2}$ (computed by weighted sum of squared residuals divided by degree of freedom: $\frac{1}{n-u}\;v^{T}\;Q_{ll}^{-1}\;v$) is equal to the prior variance-factor $\sigma_{0}^{2}$. The test quantity $T=\frac{s_{0}^{2}}{\sigma_{0}^{2}}$ follows a Fisher distribution with parameters n−u (number of condition equations minus number of parameters) and ∞. The corresponding thresholds can be found in Table 5. For the 9 parameter models the test value was about 1.0<1.5 indicating no discrepancy. Since the stochastic model is supposed to be correct for all models, the global test quantity can be treated as indicator for deterministic discrepancies. Again the distinction between deterministic and stochastic influence is important. While the non-orthogonality parameters show the biggest stochastic influence (see sensitivity analysis in Section 4), the scale parameters show the biggest deterministic influence, indicated by a heavy drop in the test quantity of the global test (765.8 for $I_{0}$ against 8.4 for $I_{1}$).

The standard deviations of bias and scale drop using 9 parameter models $I_{2}$, $I_{3}$, $I_{4}$, showing that these models might better represent the actual IMU system. Generally the scale factors are quite big, which might be a consequence of the low-cost IMU. The estimated values of models $I_{2}$ and $I_{3}$ do not differ significantly from each other. Also the standard deviations of bias and scale are quite similar. As expected from the investigations calibration improvement in Section 4.2, we are able to reduce the standard deviations of the first two non-orthogonality parameters using the extended 32 position scheme $I_{3}$. Nevertheless, the values do not change. A further increase to 48 positions ($I_{4}$) brings no enhancement concerning variance of non-orthogonality parameters. For the relevant non-orthogonality parameters $\alpha_{zx}$ and $\alpha_{zy}$ only $\alpha_{zy}$ is significantly ≠0.

### 5.2. Mounting Estimation

The results of the mounting estimation Equation (16) with the corresponding sensor model applied ($M_{i}$ is computed by applying $I_{i}$) are shown in Table 6. From the test quantity of 200 with the bias only model ($M_{0}$), we can see, that there is a clear model discrepancy. The bias and scale model $M_{1}$ almost passes the test and for the full models $M_{2}$ to $M_{4}$ the null hypotheses can be accepted. The first mounting parameter $\phi_{pb}$ does not differ significantly between $M_{1}$ and all the full models $M_{2}$ to $M_{4}$. The non-orthogonality parameter $\alpha_{zx}$ and $\phi_{pb}$ have a similar effect on leveling and tilt compensation and $\alpha_{zx}$ is insignificant different from 0, hence no difference in the estimated parameter. For the pitch mounting $\theta_{pb}$ we can see a difference of approximately $\alpha_{zy}$ between $M_{1}$ and $M_{2}$ to $M_{4}$. The accuracy of mounting parameters is also nearly the same for all full model calibrations. A calibration parameter error of 42 mgon (=three times the standard deviation) equals to an error in pole tip coordinates of 1.4 mm for 2.20 m pole length and 0.9 mm for 1.40  pole length.

### 5.3. Evaluation

Finally the complete pole tilt compensation using the IMU calibration and the mounting estimation is evaluated in an independent experiment. For this a pole length of 1.40 m (mostly used in surveying) is used to measure a known ground point with arbitrary tilts up to 50 gon. Again a three-legged clamp kept the pole static for about 2 to 5 s for each point. The total station is constantly tracking the prism and the measurements are simply time-averaged for each static period. Overall the ground point is measured 75 times.

**Figure 9:**
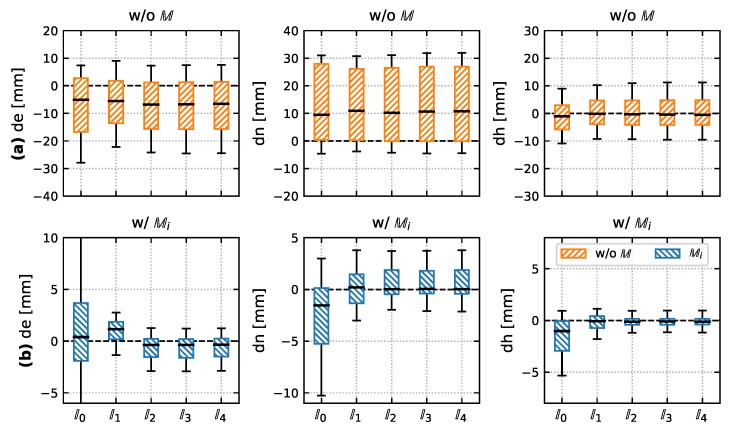
Tilt Compensation Evaluation (n=75) for different IMU sensor models $I_{0}$ to $I_{4}$. (**a**) Without Mounting. (**b**) With the corresponding mounting $M_{i}$ for $I_{i}$.

After applying the mounting $C_{b}^{p}{\left(\phi_{pb},\theta_{pb}\right)}^{T}$ and leveling $C_{b}^{n}\left(\phi_{nb},\theta_{nb}\right)$ of Equation (9), the yaw ($C_{3}{\left(\psi_{nb}\right)}^{T}$) can be computed from total station measurements and the known ground point. In fact we use ${\tilde{\psi }}_{nb}$ from ground truth ($l_{\prime }^{n}$, see below) to compute the final tilted pole vector $l^{n}$. This way, we are able to focus on the leveling parameters in our evaluation study. In addition the tilted pole vector can be computed from total station measurements $l_{\prime }^{n}$, see Equation (14). This $l_{\prime }^{n}$ would mean, that the coordinates of the ground point are correctly measured and the pole tilt compensation perfectly yields the tilted pole vector. Therefore we use $l_{\prime }^{n}$ as ground truth and analyze the differences $dl^{n}=l_{\prime }^{n}-l^{n}$. The results per component are shown in Figure 9, the numerical results for Figure 9b are listed in Table 7. Throughout the experiment, the *x*-axis of the IMU was pointing towards the *e*-axis of the total station.

Noting the different scales between the subplots (a) (without any mounting applied) and (b), it is obvious, that the application of mounting parameters is crucial for all sensor model $I_{i}$. With the according mounting parameters (Figure 9b) a Bias only sensor model ($I_{0}$) performs the worst in terms of systematic bias in pole vector components and variability. The second model ($I_{1}$) shows a bias in east component, but the scattering already reduces significantly w.r.t. $I_{0}$. The east component is the one, where errors in the second mounting parameter $\theta_{pb}$ and the non-orthogonality parameter $\alpha_{zy}$ occur (see the differences in Table 4 and Table 6 between $M_{1}$/$I_{1}$ and $M_{2}$/$I_{2}$). They are consecutive rotations about the *y*-axis. There is no significant difference between the full models $I_{2}$ to $I_{4}$ of different IMU calibration position schemes. We suppose this can have two reasons: first the estimated values of the non-orthogonality parameters $\alpha_{zx},\alpha_{zy}$ are quite similar for these models, despite the fact that aposteriori variances have improved. Secondly the mounting parameters can mask wrong non-orthogonality parameters, see Table 6.

The overall ground point (= pole tip) accuracy ($\sigma_{p}=\sqrt{\sigma_{e}^{2}+\sigma_{n}^{2}+\sigma_{h}^{2}}$), that can be achieved using the low-cost IMU with our approach is about 2.4 mm. Compared to a bias only model of accelerometer triad of 8.0 mm this is an accuracy gain of 69%, compared to a six parameter model (3.0 mm) it corresponds to a gain of 20%. The height component is the most accurate one, since it is the least sensitive one to tilt $\phi_{np},\theta_{np}$, which is composed of leveling $\phi_{nb},\theta_{nb}$ and mounting $\phi_{pb},\;\theta_{pb}$ parameters.

## 6. Conclusions

The idea of this paper is to analyze different sensor models of accelerometers triads with respect to leveling applications. In particular for pole tilt compensation, which means computational correction of a slanted position of a pole equipped with a prism. A first prototype with a consumer-grade MEMS IMU and a Raspberry Pi on a simple standard pole from engineering geodesy has been developed. The formalism for pole tilt compensation is successfully validated with this prototype. For that the unknown parameter in this formalism must first be estimated with an appropriate calibration routine, elaborated in this contribution.

The core part of this contribution is related to the question of important parameters in this model. We utilize methods of variance based sensitivity analysis to identify main contributors to the final theoretical uncertainty of the target quantities, for example, tilt angles or pole vector components. We have shown, that the non-orthogonality parameters of the z-axis accelerometer $\alpha_{zy}$ and $\alpha_{zx}$ are the most important both in terms of uncertainty contribution as well as deterministic error introduction. Using this derived insights an iterative application oriented calibration design is proposed to improve the estimated calibration parameters. This concept is formulated to be easily portable to any other calibrations tasks to improve in terms of result accuracy, economics and time saving. Overall sensitivity analysis is useful, both to identify important parameters and to examine how these can be improved.

First the proposed two-part calibration routine consisting of an accelerometer triad calibration using an industrial robot and a mounting estimation with the involved total station is executed. The final evaluation experiment proofs the validity of the proposed models and calibration procedures. With an exact known third orientation angle $\psi_{np}$ we are able to achieve a ground point accuracy of 2.4 mm. The experiment also reveals the necessity of a 9 parameter model consisting of three biases $b_{f}$, three scales $s_{f}$ and three non-orthogonality $\alpha_{f}$ parameters. Finally three different position schemes for IMU calibration are tested, but showed no performance improvement, it is therefore reasonable to assume that the 24 position scheme from Reference [26] is sufficient.

Future plans are to repeat the experiments with an industrial grade MEMS IMU and compare the findings between IMUs of different noise level. One can also think about reformulating the compensation model using quaternion attitude representation to overcome singularities inherent in Euler angles and allow the pole for a full-dome probing device. Also for real world application future research has to focus on yaw ($\psi_{np}$) estimation, since its uncertainty accounts for up to 75% of the pole components uncertainty.

## Figures and Tables

**Table 1:** Related research of accelerometer triad error models, sorted by ascending date of first publication.

Authors	Model Parameters	Positions
[23]	Bias and Scale	6
[16,17,24,25]	Bias, Scale and Non-orthogonalities	18
[22]	Bias, Scale, Non-orthogonalities and Cross-axis sensitivities	18 and 24
[19]	Bias, Scale and Non-orthogonalities	9
[26]	Bias, Scale, Nonlinear Scale and Non-orthogonalities	24
[27,28]	Bias, Scale and Non-orthogonalities	36–50
[21]	Bias, Scale, Non-orthogonalities and Misalignment	30

**Table 2:** Estimated biases of IMU calibration.

Model/Scheme	$b_{x}$ [$\mathrm{mm}\;s^{-2}$]	$\sigma_{b_{x}}$ [$\mathrm{mm}\;s^{-2}$]	$b_{y}$ [$\mathrm{mm}\;s^{-2}$]	$\sigma_{b_{y}}$ [$\mathrm{mm}\;s^{-2}$]	$b_{z}$ [$\mathrm{mm}\;s^{-2}$]	$\sigma_{b_{z}}$ [$\mathrm{mm}\;s^{-2}$]
Bias only 24 pos ($I_{0}$)	−329.62	40.49	−204.85	40.78	−262.85	38.98
Bias and Scale 24 pos ($I_{1}$)	−348.63	4.33	−198.72	4.25	−263.34	4.08
Full Model 24 pos ($I_{2}$)	−349.81	1.49	−199.30	1.47	−263.59	1.40
Full Model 32 pos ($I_{3}$)	−348.99	1.15	−199.03	1.15	−263.51	1.02
Full Model 48 pos ($I_{4}$)	−350.63	0.94	−198.96	0.92	−263.64	0.86

**Table 3:** Estimated scale factor of IMU calibration.

Model/Scheme	$s_{x}$ [ppm]	$\sigma_{s_{x}}$ [ppm]	$s_{y}$ [ppm]	$\sigma_{s_{y}}$ [ppm]	$s_{z}$ [ppm]	$\sigma_{s_{z}}$ [ppm]
Bias only 24 pos ($I_{0}$)	n.a.	n.a.	n.a.	n.a.	n.a.	n.a.
Bias and Scale 24 pos ($I_{1}$)	19,711	509	−13,059	498	−5539	475
Full Model 24 pos ($I_{2}$)	19,683	175	−12,914	172	−5553	163
Full Model 32 pos ($I_{3}$)	19,710	142	−12,860	140	−5504	129
Full Model 48 pos ($I_{4}$)	19,087	112	−13,499	108	−6134	100

**Table 4:** Estimated non-orthogonality parameters of IMU calibration.

Model/Scheme	$\alpha_{\mathrm{zx}}$ [mgon]	$\sigma_{\alpha_{\mathrm{zx}}}$ [mgon]	$\alpha_{\mathrm{zy}}$ [mgon]	$\sigma_{\alpha_{\mathrm{zy}}}$ [mgon]	$\alpha_{\mathrm{yz}}$ [mgon]	$\sigma_{\alpha_{\mathrm{yz}}}$ [mgon]
Bias only 24 pos ($I_{0}$)	n.a.	n.a.	n.a.	n.a.	n.a.	n.a.
Bias and Scale 24 pos ($I_{1}$)	n.a.	n.a.	n.a.	n.a.	n.a.	n.a.
Full Model 24 pos ($I_{2}$)	−10.8	28.0	−172.3	27.4	−310.4	29.2
Full Model 32 pos ($I_{3}$)	−11.2	17.2	−171.6	16.8	−309.1	25.3
Full Model 48 pos ($I_{4}$)	5.4	16.8	−162.4	16.5	−329.3	18.3

**Table 5:** Global test of adjustment for different sensor models.

Model/Scheme	Parameters	Threshold []	$T(H_{0})$ []	Acceptance
Bias only 24 pos ($I_{0}$)	$b_{f}$	1.56	765.8	no
Bias and Scale 24 pos ($I_{1}$)	$b_{f}$, $s_{f}$	1.60	8.4	no
Full Model 24 pos ($I_{2}$)	$b_{f}$, $s_{f}$, $\alpha_{f}$	1.67	1.2	yes
Full Model 32 pos ($I_{3}$)	$b_{f}$, $s_{f}$, $\alpha_{f}$	1.53	1.0	yes
Full Model 48 pos ($I_{4}$)	$b_{f}$, $s_{f}$, $\alpha_{f}$	1.40	0.9	yes

**Table 6:** Mounting parameters with corresponding test quantity of adjustments global test. Threshold for $H_{0}$ acceptance is about 1.4.

Model/Scheme	$\phi_{\mathrm{pb}}$ [gon]	$\sigma_{\phi_{\mathrm{pb}}}$ [gon]	$\theta_{\mathrm{pb}}$ [gon]	$\sigma_{\theta_{\mathrm{pb}}}$ [gon]	$T(H_{0})$ []
Bias only (24) $M_{0}$	1.4602	0.1610	−0.9557	0.1770	200.30
Bias and Scale (24) $M_{1}$	1.2526	0.0143	−0.6824	0.0154	1.56
Full Model (24) $M_{2}$	1.2465	0.0128	−0.8364	0.0138	1.25
Full Model (32) $M_{3}$	1.2620	0.0130	−0.8243	0.0141	1.31
Full Model (48) $M_{4}$	1.2639	0.0129	−0.8225	0.0139	1.28

**Table 7:** Corresponding results (units of [mm]) in numbers for Figure 9b.

	Mean(de)	Mean(dn)	Mean(dh)	rms(de)	rms(dn)	rms(dh)
$I_{0}$	0.8	−2.5	−1.8	5.6	4.8	3.1
$I_{1}$	1.0	0.2	−0.2	1.7	2.2	1.0
$I_{2}$	−0.6	0.5	−0.1	1.4	1.8	0.7
$I_{3}$	−0.6	0.5	−0.1	1.4	1.8	0.7
$I_{4}$	−0.6	0.5	−0.1	1.4	1.8	0.7

## Abbreviations

The following abbreviations are used in this manuscript:

GNSS	Global Navigation Satellite System
IMU	Inertial Measurement Unit
MEMS	Micro-Electro-Mechanical Systems
VBSA	Variance Based Sensitivity Analysis
VCM	Variance Covariance Matrix

## Appendix A. Definition of Rotation Matrices

Throughout this contribution a *Tait-Bryan* Euler angles definition of sequence *z-y-x* with mathematically positive rotation direction is used.

(A1) $$\begin{array}{cc} C_{\beta }^{\alpha } & =C_{1}\left(\phi_{\beta \alpha }\right)\;C_{2}\left(\theta_{\beta \alpha }\right)\;C_{3}\left(\psi_{\beta \alpha }\right)=\\ \; & =\left[\begin{array}{ccc} 1 & 0 & 0\\ 0 & \cos\phi_{\beta \alpha } & \sin\phi_{\beta \alpha }\\ 0 & -\sin\phi_{\beta \alpha } & \cos\phi_{\beta \alpha } \end{array}\right]\left[\begin{array}{ccc} \cos\theta_{\beta \alpha } & 0 & -\sin\theta_{\beta \alpha }\\ 0 & 1 & 0\\ \sin\theta_{\beta \alpha } & 0 & \cos\theta_{\beta \alpha } \end{array}\right]\left[\begin{array}{ccc} \cos\psi_{\beta \alpha } & \sin\psi_{\beta \alpha } & 0\\ -\sin\psi_{\beta \alpha } & \cos\psi_{\beta \alpha } & 0\\ 0 & 0 & 1 \end{array}\right] \end{array}$$

Since the euler angles $\psi_{\beta \alpha }$ describe the orientation of the object frame α with respect to the reference frame β, the rotation from α to β is very often needed to convert between attitude representations:

(A2) $$\begin{array}{cc} C_{\alpha }^{\beta } & ={C_{\beta }^{\alpha }}^{T}=C_{3}{\left(\psi_{\beta \alpha }\right)}^{T}\;C_{2}{\left(\theta_{\beta \alpha }\right)}^{T}\;C_{1}{\left(\phi_{\beta \alpha }\right)}^{T}=\\ \; & =\left[\begin{array}{ccc} c\theta_{\beta \alpha }\;c\psi_{\beta \alpha } & -c\phi_{\beta \alpha }\;s\psi_{\beta \alpha }+s\phi_{\beta \alpha }\;s\theta_{\beta \alpha }\;c\psi_{\beta \alpha } & s\phi_{\beta \alpha }\;s\psi_{\beta \alpha }+c\phi_{\beta \alpha }\;s\theta_{\beta \alpha }c\psi_{\beta \alpha }\\ c\theta_{\beta \alpha }\;s\psi_{\beta \alpha } & c\phi_{\beta \alpha }\;c\psi_{\beta \alpha }+s\phi_{\beta \alpha }\;s\theta_{\beta \alpha }\;s\psi_{\beta \alpha } & -s\phi_{\beta \alpha }\;c\psi_{\beta \alpha }+c\phi_{\beta \alpha }\;s\theta_{\beta \alpha }\;s\psi_{\beta \alpha }\\ -s\theta_{\beta \alpha } & s\phi_{\beta \alpha }\;c\theta_{\beta \alpha } & c\phi_{\beta \alpha }\;c\theta_{\beta \alpha } \end{array}\right]=\\ \; & =C_{\alpha }^{\beta }\left(\phi_{\beta \alpha },\theta_{\beta \alpha },\psi_{\beta \alpha }\right)\;, \end{array}$$

where cγ is short for cosγ and sγ is short for sinγ. If we use the notation $C_{\alpha }^{\beta }$ or $C_{\alpha }^{\beta }\left(\phi_{\beta \alpha },\theta_{\beta \alpha },\psi_{\beta \alpha }\right)$, the full rotation matrix (A2) is meant. If for example, $C_{\alpha }^{\beta }\left(\phi_{\beta \alpha },\theta_{\beta \alpha }\right)$ is written, it is short for $C_{\alpha }^{\beta }\left(\phi_{\beta \alpha },\theta_{\beta \alpha },\psi_{\beta \alpha }=0\right)$, which is in fact:

(A3) $$\begin{array}{cc} C_{\alpha }^{\beta }\left(\phi_{\beta \alpha },\theta_{\beta \alpha }\right) & =C_{3}{\left(\psi_{\beta \alpha }=0\right)}^{T}\;C_{2}{\left(\theta_{\beta \alpha }\right)}^{T}\;C_{1}{\left(\phi_{\beta \alpha }\right)}^{T}=\\ \; & =\underset{3\times 3}{I}\;C_{2}{\left(\theta_{\beta \alpha }\right)}^{T}\;C_{1}{\left(\phi_{\beta \alpha }\right)}^{T}=C_{2}{\left(\theta_{\beta \alpha }\right)}^{T}\;C_{1}{\left(\phi_{\beta \alpha }\right)}^{T} \end{array}$$

## Appendix B. Adjustment Sensitivity Analysis Algorithm

Starting from a set of implicit equations ($L_{0}$) and given approximate values of the parameters ($x_{0}$) the corresponding observations $l_{0}$ can be computed using a simulation framework. Also define a set of implicit candidate equations $L_{c}$.

The design matrix A contains the partial derivatives of the functional model φ w.r.t. the parameters x and the observation matrix B hold the partial derivative w.r.t. the observations l. For details refer to for example, Reference [37].

**Algorithm A1** Adjustment Sensitivity Algorithm
Initialize $\Sigma_{ll}^{0}$, $l^{0}$ Compute $A_{0}$, $B_{0}$ from $l^{0}$, $x^{0}$ $\Sigma_{\hat{x}\hat{x}}^{0}\leftarrow {\left(A_{0}^{T}{\left(B_{0}\;\Sigma_{ll}^{0}\;B_{0}^{T}\right)}^{-1}A_{0}\right)}^{-1}$ Initialize I with dimensions $\mathrm{len}\left(L_{c}\right)\times \mathrm{len}\left(x\right)$ **for all** $\left(l,\mathrm{counter}\;i\right)\;\mathrm{in}\;L_{c}$ **do** $l_{i}\leftarrow {\left[\begin{array}{cc} l_{0}^{T} & l^{T} \end{array}\right]}^{T}$, $\Sigma_{ll}^{i}\leftarrow \left[\begin{array}{cc} \Sigma_{ll}^{0} & 0\\ 0 & \Sigma_{ll} \end{array}\right]$ Compute A, B from $l_{i}$, $x^{0}$ $A_{i}\leftarrow {\left[\begin{array}{cc} A_{0}^{T} & A^{T} \end{array}\right]}^{T}$, $B_{i}\leftarrow \left[\begin{array}{cc} B_{0} & 0\\ 0 & B \end{array}\right]$ $\Sigma_{\hat{x}\hat{x}}^{i}\leftarrow {\left(A_{i}^{T}{\left(B_{i}\;\Sigma_{ll}^{i}\;B_{i}^{T}\right)}^{-1}A_{i}\right)}^{-1}$ $I\left[i,:\right]\leftarrow 1-\frac{\mathrm{diag}(\Sigma_{xx}^{i})}{\mathrm{diag}(\Sigma_{xx}^{0})}$ **end for**

Now I holds the relative improvement in parameter variances for each equation candidate in $L_{c}$ in the rows. Look at the corresponding column *k* for the parameter(s) to be improved and choose the maximum:

(A4) $$\mathrm{equation}\;\mathrm{to}\;\mathrm{add}\;L_{c}\left\{i\right\}\leftarrow \underset{i}{\arg\;\max}\;I\left[:,k\right]\;,$$

and add it to the initial set $L_{0}$.

Finally repeat the process until an exit criterion is met, for example, maximum number of condition equations or minimal improvement achievable. Note that the current version of this algorithm does not consider correlation (off-diagonal elements of $\Sigma_{\hat{x}\hat{x}}$).
